# The structure of personality in Parkinson’s disease and the effects of age, years since diagnosis, and impulsivity

**DOI:** 10.7717/peerj.20725

**Published:** 2026-02-19

**Authors:** Stefano Vicentin, Lavinia Chiriatti, Giorgia Cona

**Affiliations:** 1 Institute for Psychology, Clinical Psychology and Psychotherapy in Childhood and Adolescence, Universität Osnabrück, Osnabrück, Lower Saxony, Germany; 2 Department of General Psychology, University of Padua, Padua, Italy; 3 Padua Neuroscience Center, University of Padua, Padua, Italy

**Keywords:** Parkinson’s Disease, Personality, Impulsive-Compulsive disorder, Aging

## Abstract

**Background:**

Parkinson’s disease (PD) is a progressive neurodegenerative disorder primarily characterized by motor and cognitive symptoms. However, emerging evidence suggests that personality alterations may also be present, potentially affecting patients’ quality of life and clinical outcomes. Prior studies have identified patterns such as lower openness and extraversion and higher neuroticism in PD patients, although findings have been inconsistent. This study aimed to investigate the structural organization of personality in PD using a network-based approach, and to examine the influence of age, sex, disease duration, and impulsive-compulsive symptoms on personality traits.

**Methods:**

A total of 237 PD patients (aged 45–86) completed the HEXACO Adjective Scale (HAS), assessing six personality traits: Honesty-Humility (H), Emotionality (E), Extraversion (X), Agreeableness (A), Conscientiousness (C), and Openness (O). Impulsive-compulsive behaviors were assessed using the Questionnaire for Impulsive-Compulsive Disorders in Parkinson’s Disease–Rating Scale (QUIP-RS). Personality structure was analyzed *via* Exploratory Graph Analysis (EGA), a network model that identifies item clusters based on conditional dependencies. Multivariate multiple linear regression was used to test the effects of demographic and clinical variables on trait expression.

**Results:**

EGA identified seven item-level communities. Traits E, X, and C formed coherent and distinct clusters, while items from A and H tended to cluster based on item polarity (positive *vs.* negative wording) rather than theoretical trait boundaries. O items split into two distinct communities, one composed of the items from the *Unconventionality* facet, and the other encompassing the remaining O items. At the trait level, HEXACO dimensions grouped into two higher-order clusters: a Cooperativity–Integrity community (H, A, C) and an Engagement community (E, X, O). Regression analyses showed that higher ICD symptoms predicted lower levels of H, A, and C; longer disease duration was associated with lower C, and sex showed significant differences in E.

**Conclusions:**

These findings reveal subtle but systematic alterations in the structural organization of personality traits in PD. Specifically, we observed a polarity-based overlap between H and A, possibly reflecting age-related convergence of the two traits—consistent with a recent study reporting similar effects in healthy aging—and a bifurcation within O centered on the Unconventionality facet, a trait often considered idiopathic in PD, even in its prodromal stages. These personality signatures may contribute to a refined clinical profiling of PD patients and support the value of incorporating personality assessment into personalized care approaches.

## Introduction

Parkinson’s disease (PD) is a progressive neurodegenerative disorder primarily characterized by motor symptoms such as tremors, rigidity, and bradykinesia. Alongside these hallmark impairments, PD also entails a wide array of non-motor symptoms, including cognitive decline—particularly in executive functioning and memory (Svenningsson et al., 2012; Watson & Leverenz, 2010)—as well as behavioral and affective changes, such as increased impulsivity, depressive symptoms, and personality alterations (Ishihara & Brayne, 2006a; Callesen et al., 2014). Despite growing attention to personality changes in PD, evidence remains inconclusive and fragmented.

Historically, research on personality in PD has followed varied aims and frameworks. One early line of investigation explored what was described as the Premorbid Parkinsonian Personality, hypothesizing a stable profile—marked by traits such as industriousness, inflexibility, cautiousness, conventionality, and low impulsivity—preceding diagnosis (Ishihara & Brayne, 2006b). According to this hypothesis, individuals developing PD tended to exhibit a set of personality traits before the appearance of motor symptoms and diagnosis. However, this theory has been challenged due to inconsistent findings and methodological limitations, including retrospective assessments and heterogeneous instruments (Poletti & Bonuccelli, 2012a). Other studies focused on identifying personality traits that distinguish PD patients from healthy individuals or those with other neurodegenerative conditions, aiming to detect hallmark personality alterations associated with PD and, potentially, to gain insight into the neurobiological underpinnings of personality by examining overlaps with the neuropathology of PD (Santangelo et al., 2017).

The heterogeneity of goals in the study of PD-related personality characteristics has also been mirrored by the variety of theoretical frameworks employed. Research has mostly focused on Cloninger’s Psychobiological Model of Personality (Cloninger, 1987) and the Five Factor Model (FFM) (Costa & McCrae, 1992; McCrae & John, 1992). Cloninger’s model, operationalized through instruments such as the Tridimensional Personality Questionnaire, posits that personality is shaped by neurobiological systems and comprises dimensions such as novelty seeking, harm avoidance, and reward dependence. Studies using this model have consistently shown that PD patients tend to exhibit lower novelty seeking and higher harm avoidance compared to healthy controls (Poletti & Bonuccelli, 2012a). The FFM, on the other hand, is trait-based model that includes five dimensions: neuroticism, extraversion, openness to experience, agreeableness, and conscientiousness. Several studies have found associations between PD and elevated neuroticism, along with lower levels of extraversion and conscientiousness (Baig et al., 2017; Baig et al., 2019; de Souza Arten & Hamdan, 2023). A meta-analysis combining findings from both frameworks confirmed consistent increases in harm avoidance and neuroticism and decreases in novelty seeking, extraversion, and openness (Santangelo et al., 2018). Although these findings are robust, their specificity to PD remains debated, as similar patterns have been observed in healthy aging and other clinical populations (D’Iorio et al., 2018; Trouillet & Gana, 2008). Moreover, the distinction between premorbid personality features and disease-related changes remains unclear.

More recently, the HEXACO model has gained traction as a refined framework in personality research (Ashton & Lee, 2009). This model describes six broad dimensions, or traits: Honesty-Humility (H), Emotionality (E), Extraversion (X), Agreeableness (A), Conscientiousness (C), and Openness (O; Ashton & Lee, 2007). Hence, this framework can be seen as an extension of the FFM, with the traditional A domain split to separately capture traits related to sincerity, fairness, and modesty (H) and those related to patience and forgiveness (A), and the E dimension partially overlapping with the neuroticism trait, but with greater emphasis on emotional attachment, sentimentality, and dependence rather than general emotional instability. These distinctions enhance the capacity of the HEXACO model to capture socially relevant and moral behaviors and may offer increased sensitivity to subtle personality alterations in clinical contexts. So far, research using the HEXACO model has primarily focused on healthy adults, especially in the context of lifespan development. For instance, Gobbo, Trapattoni & Romano (2025) investigated personality structure in healthy older adults and found evidence of a partial reorganization of the six HEXACO traits, including increased levels of H and a tendency for H and A items to cluster together based on emotional valence rather than by trait content. These findings suggest that personality structure may change across the lifespan, with trait configuration influenced by broader socio-emotional dynamics. Investigations using the HEXACO framework in clinical populations, on the other hand, remain comparatively underexplored. A notable contribution in the broader mental health context is the meta-analysis by Pletzer, Thielmann & Zettler (2024), which reported meaningful associations between personality traits—particularly X—and psychological well-being. Together, these findings highlight the importance of examining personality structure in populations characterized by neurodegeneration and socio-emotional adaptation demands, such as individuals with PD.

To address this gap, the present study investigates the structure of personality in individuals with PD using the HEXACO framework. Rather than focusing solely on trait level differences, we examined whether the structural organization of personality—defined as the configuration and interrelations among traits—differs from patterns typically observed in healthy adults. In addition, we aimed to explore how demographic (age, sex, education) and clinical (years since diagnosis, impulsive-compulsive symptoms) factors are associated with personality traits. To this end, the effect of these variables on personality as a whole and on each HEXACO trait was analyzed. Based on previous work, we expected age to reflect general socio-emotional processed associated with healthy aging (Gobbo, Trapattoni & Romano, 2025), and years since diagnosis to capture PD-specific progression effects (Raket et al., 2022). We also expected sex differences to be most pronounced in E (Hu et al., 2018; Szewczyk-Krolikowski et al., 2014). Finally, in line with prior evidence linking impulsive-compulsive behaviors to reduced self-regulatory tendencies, we anticipated that ICD severity would be negatively associated with C (Mao et al., 2018; Poletti & Bonuccelli, 2012b).

## Materials & Methods

### Participants

Data were collected from individuals diagnosed with PD who voluntarily participated in the study. Recruitment was conducted both in person—through scheduled appointments with hospitals and local associations in the Veneto region (Italy)—and online, by promoting the study in Facebook groups and *via* email outreach to PD support groups, associations, and facilities. Although the questionnaires were primarily administered online, researchers provided assistance to participants with limited digital literacy whenever possible and upon request.

Exclusion criteria included the presence of other neurological or psychiatric disorders and failure to complete the demographic questionnaire (age, education, and years since diagnosis) or the full HEXACO Adjective Scale. Participants who did not meet these criteria were excluded from the analyses.

### Procedure

Data were collected using Qualtrics, an online platform to develop and administer surveys (Qualtrics, 2024), as part of a research project approved by the Ethical Committee of the University of Padova (Protocol number: 623-b) and funded by the Italian Ministry of University and Research (MUR) (Project: SCOPERTA; Research Project of National Interest (PRIN), grant number 2022BNMZJC). The study was designed and conducted in accordance with the guidelines of the Declaration of Helsinki. Written informed consent was obtained from all participants prior to participation. The study also adheres to the PECANS guidelines (Costa et al., 2025). The experimental protocol consisted of three main sections. First, participants completed a demographic questionnaire collecting data on age, sex, education, and years since diagnosis. Second, the HEXACO Adjective Scale (HAS) was administered to assess personality traits (Romano et al., 2023). The HAS is a novel tool designed to measure the six HEXACO personality dimensions using adjectives rather than full statements. Compared to the standard HEXACO-PI-R questionnaire (Ashton & Lee, 2009), the HAS has demonstrated psychometric properties better suited for use in older populations (Gobbo, Trapattoni & Romano, 2025). It is also more efficient and accessible, offering a streamlined format that reduces the likelihood of misinterpretation. The third and final section consisted of the Questionnaire for Impulsive-Compulsive Disorders in Parkinson’s Disease–Rating Scale (QUIP-RS), a standardized instrument used to assess levels of impulsivity and compulsivity and to evaluate the presence and severity of Impulse Control Disorder (ICD)-related symptoms, such as gambling, hypersexuality, and compulsive shopping, in individuals with PD (Maggi et al., 2024). The questionnaire is published under a Creative Commons (CC BY 4.0) license, permitting its use and reproduction in this study.

### Statistical analyses

All analyses were conducted using R (version 2025.5.0.496; R Core Team, 2025). The structure of personality was examined using Exploratory Graph Analysis (EGA) method (Golino & Epskamp, 2017). EGA is a network-based approach that allows to examine the structure of high-dimensional data by treating individual variables as nodes and estimating associations between them as edges. This method enables the identification of communities, or clusters of items that tend to co-occur, offering insight into the latent structure of personality traits (Hudson & Alexander, 2024). By estimating all item associations simultaneously, EGA allows the examination of how traits cluster, separate, or reorganize in non-normative ways in different populations, revealing patterns that are not visible from mean trait scores alone. Unlike traditional factor analysis, which requires specifying or testing predefined latent dimensions, EGA allows the data to reveal how items naturally group, making it possible to detect alternative structural configurations when they occur. This feature is particularly relevant in clinical populations such as PD, where personality organization may diverge from patterns typically observed in healthy adults. While no standard procedure exists for calculating power or sample size in network analysis, a sample size of at least 200 participants is generally considered sufficient to detect stable network structures. Given the large number of items in the HAS (60 nodes), LASSO regularization was applied to estimate the network (Epskamp, Borsboom & Fried, 2018). To assess the stability and replicability of the identified communities, we performed a nonparametric bootstrapping with 5,000 iterations. This procedure estimates how consistently the same community configuration re-emerges across resampled datasets, providing an index of structural stability.

Two distinct network analyses were performed. The first EGA was performed at the item level, focusing on the emergence of communities from participants’ responses and testing whether these clusters aligned with the six expected HEXACO traits. A second network analysis was performed at the trait level, investigating the associations among the six aggregated personality dimensions and whether they formed distinct communities.

The second phase of analysis examined how demographic and clinical variables influenced personality trait expression. A multivariate multiple linear regression was performed to evaluate the effects of age, years since diagnosis, sex, education level (ISCED), and impulsive-compulsive symptom severity (ICD score) on the six HEXACO dimensions. Age and years since diagnosis were included simultaneously to distinguish effects related to general aging from those reflecting disease progression (Raket et al., 2022). Sex was included due to previously documented differences in both clinical presentation and personality profiles in PD (Hu et al., 2018; Szewczyk-Krolikowski et al., 2014). Education was considered as a covariate related to cognitive reserve and openness-related personality characteristics. Finally, ICD severity was included due to its high prevalence in PD, particularly in individuals treated with dopaminergic medication (Spencer & Anderton, 2024; Voon et al., 2010). We assessed both global effects on the overall personality profile and specific effects on each HEXACO trait. An a priori power analysis using G*Power (Faul et al., 2007) indicated the minimum sample size for the planned multivariate regression model in 146 participants (*α* = .05, power = .80).

## Results

### Demographics

A total of 249 individuals with PD were initially recruited. Of these, 12 were excluded due to missing demographic or HAS data, or because they reported additional neurological or psychiatric conditions. The final sample included in the network analyses consisted of 237 participants (mean age = 64.90 ± 9.10 years, range = 45–86; 125 females). The average number of years since diagnosis was 7.36 ± 5.73. Educational level, assessed using the ISCED scale, averaged 3.66 ± 1.31.

Among these 237 participants, 25 did not complete the QUIP-RS questionnaire and were therefore excluded from the regression analysis. Therefore, the final sample for this second analysis consisted of 202 participants (mean age = 64.81 ± 8.70 years; range = 45–85; 106 females; mean years since diagnosis = 7.36 ± 5.64; mean ISCED = 3.65 ± 1.32;). This sample size exceeded the minimum required for adequate power as indicated by the a priori power analysis (*N* = 146).

### Network analysis

**Figure 1:**
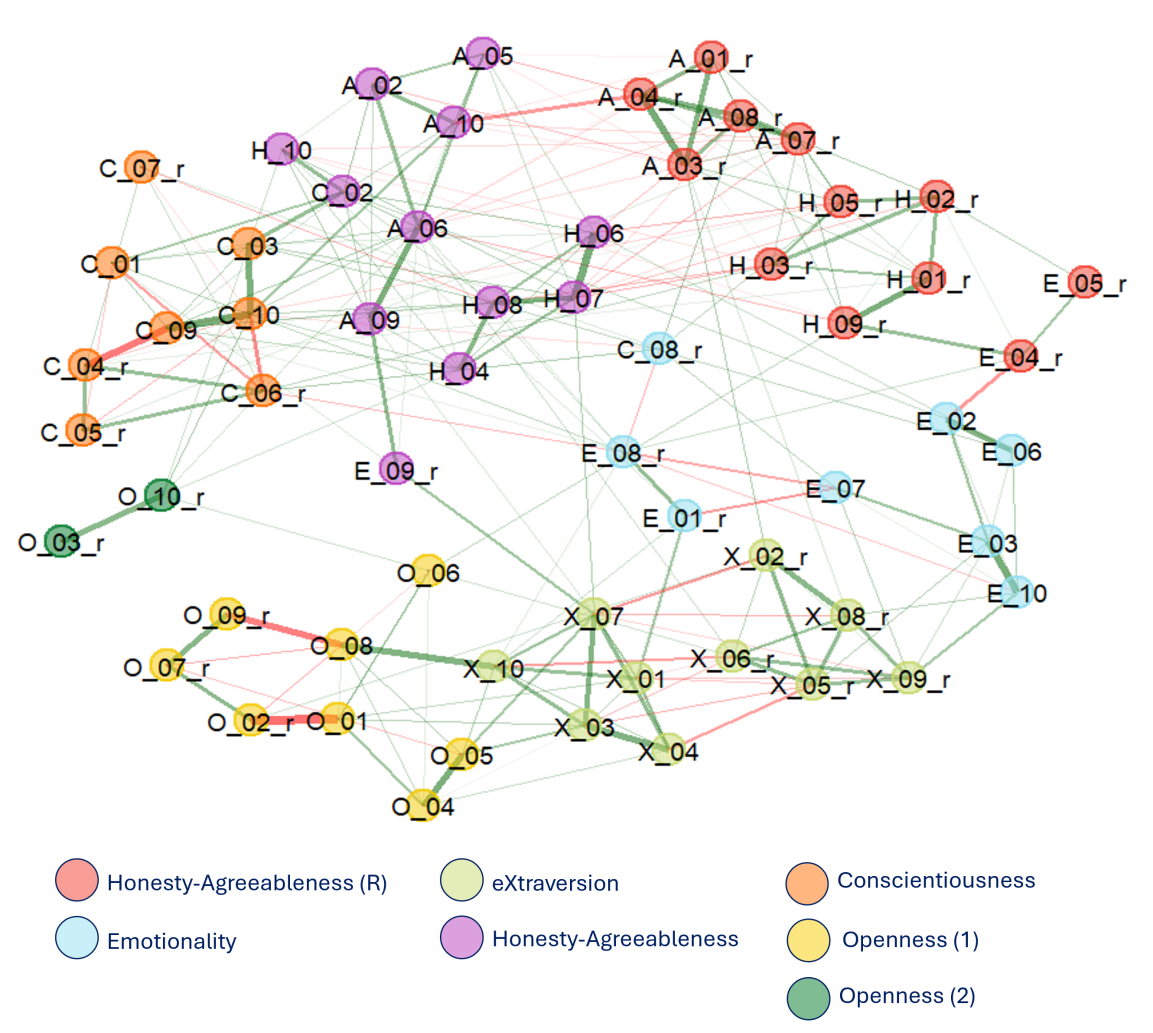
Network structure emerging from the 60 HAS items. Items are labeled by progressive number; items ending in “_r” indicate reversed-keyed adjectives.

The network arising from the EGA analysis consisted of 238 edges (mean weight = .011), yielding a density of 0.134, indicating a sparsely connected but structured network. The 60 HAS items were found to cluster into seven distinct communities, broadly consistent with the six core HEXACO traits (Fig. 1). Specifically, items associated with E, X, C, and O formed relatively coherent and trait-specific communities. However, the two O items forming the Unconventionality facet—*Traditional and Conventional*—emerged as a separate, seventh community. This suggests strong collinearity between the two items, as well as weaker associations with the other O facets. Finally, the H and A items did not form separate specific clusters but were instead grouped into two communities organized along valence polarity: a community composed of the direct items, characterized by a positive valence (*e.g.*, *Honest, Peaceful*), and the other formed by the reversed (negatively keyed) items (*e.g.*, *Hypocritical, Aggressive*). In other words, A and H items (adjectives) resulted to be more correlated depending on their valence polarity, rather than by their theoretical trait assignments, an effect not found in the other traits.

To assess stability, a bootstrap analysis (5,000 iterations) was conducted. The seven-community configuration was the most frequent outcome (30.2% of iterations), followed closely by a six-community solution (25.6%), indicating comparable stability between the two. The smallest O community (composed of the two items *Traditional* and *Conventional*) showed low replicability, appearing in only 34% of replications, suggesting limited stability of this cluster.

Regarding the valence-based H–A communities, replicability varied systematically between traits within each cluster. In the cluster composed of negatively keyed items, H items showed relatively high replicability (above 80%), while A items replicated less consistently, clustering together in approximately 60% of iterations. A similar pattern emerged in the positively keyed cluster, where A items exhibited high stability (80–90%), but H items replicated less robustly, with about 55% consistency. These findings suggest that, although the valence-driven reorganization is a prominent feature of the network, it remains partially unstable and asymmetrical across traits, with H items showing greater variability in clustering than A items.

**Figure 2:**
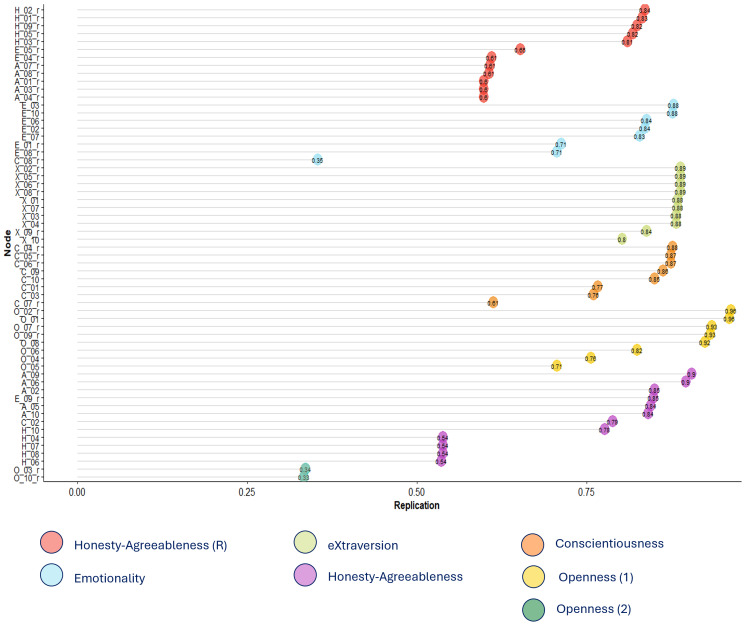
Replications of the 60 HAS items in the network analysis. Values closer to 1 represent higher replications within the same cluster/community.

Figure 2 displays the replications stability of each item in the bootstrap analysis.

**Figure 3:**
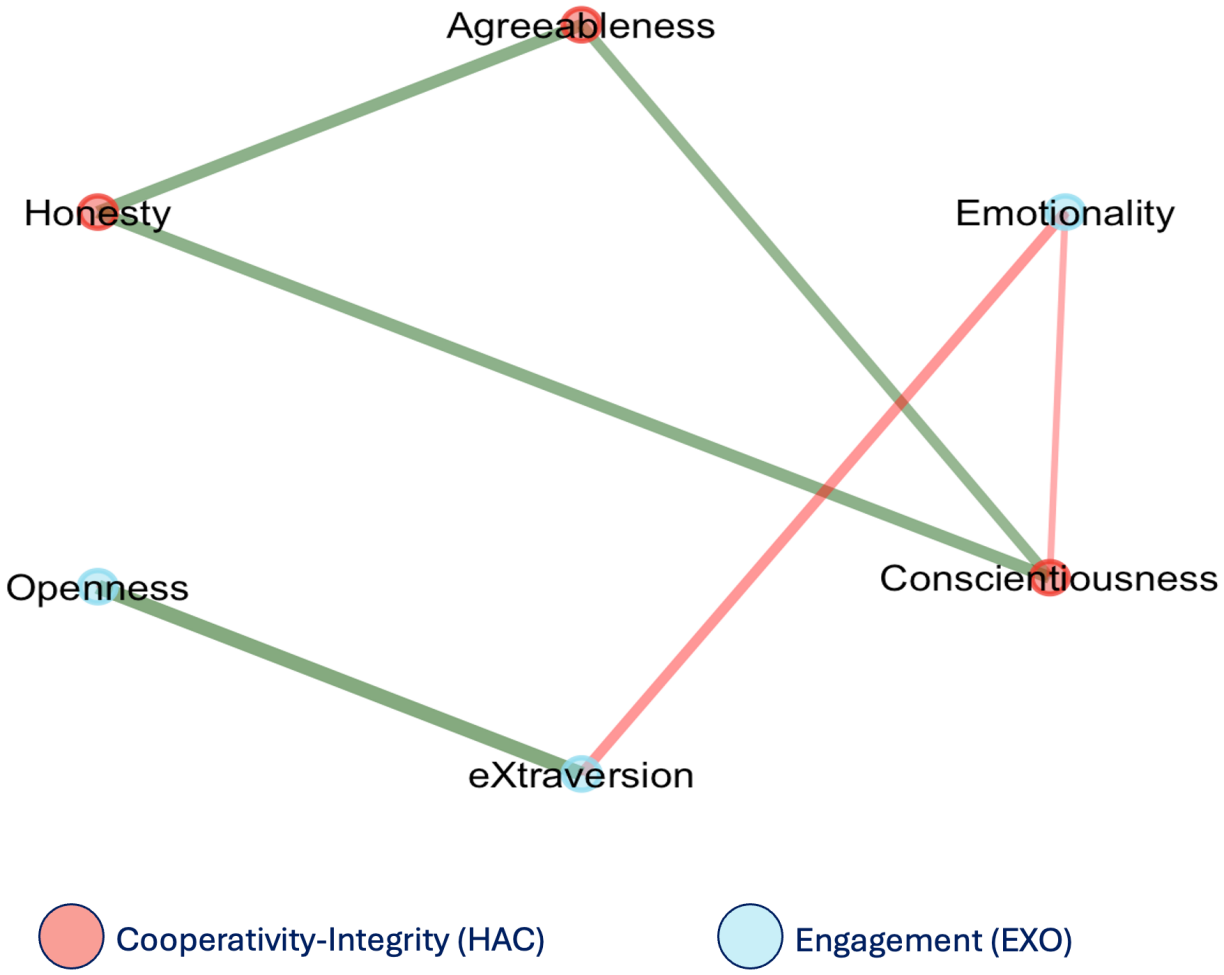
Network structure emerging from the six HEXACO traits.

**Figure 4:**
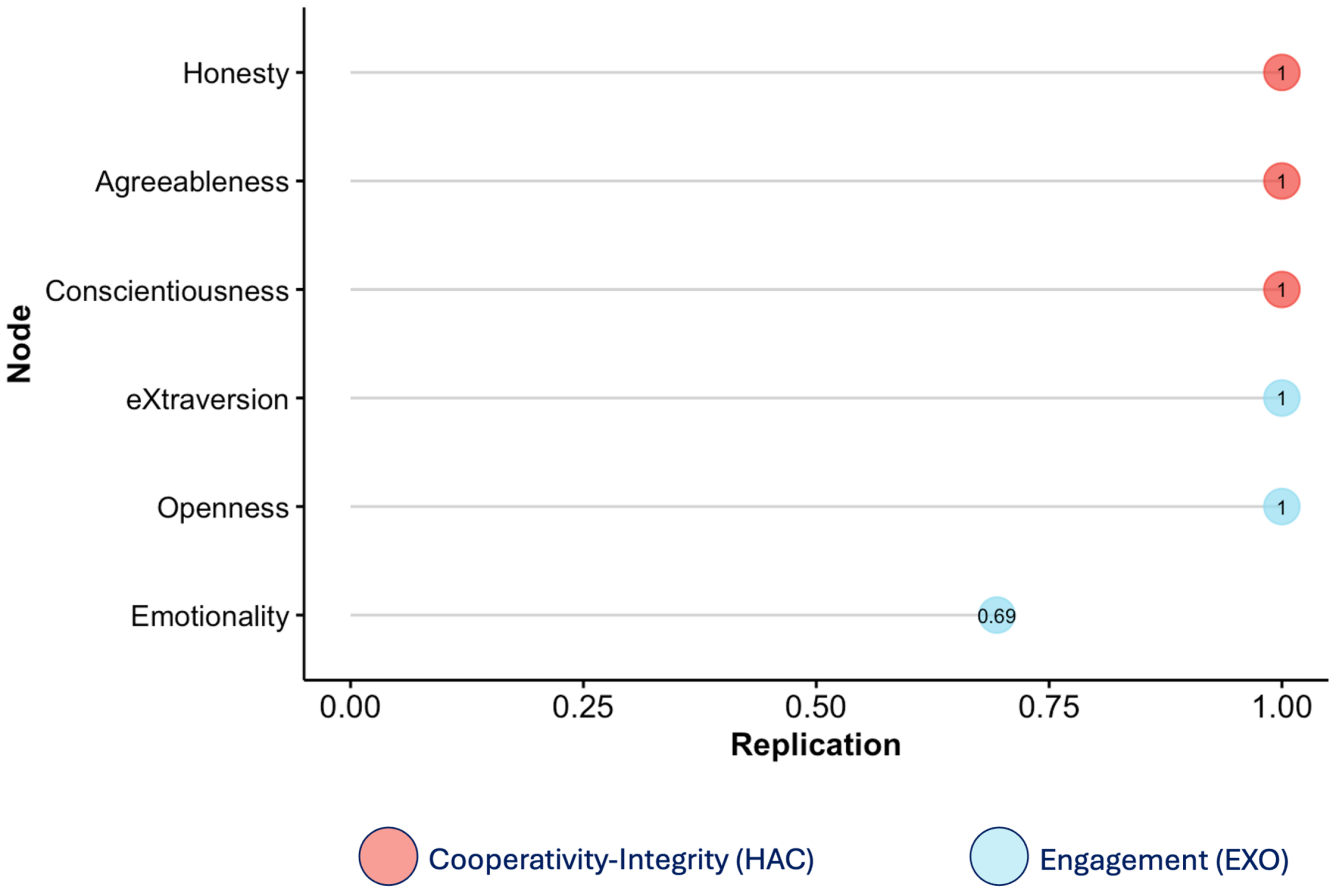
Replications of the six HEXACO traits scores in the network analysis. Values closer to 1 represent higher replications within the same cluster/community.

A second EGA was performed at the trait level, focusing on the interrelations among the six HEXACO dimensions (Fig. 3). This resulted in a network composed of six edges connecting the six HEXACO personality traits (density = .400, mean weight = .010), indicating a moderately connected network. The nodes (traits) clustered into two separate communities, one composed of H, A, and C (labelled as the “Cooperativity-Integrity” community) and the other by E, X, and O (“Engagement” community). This two-cluster solution replicated strongly across bootstraps. All traits remained in their respective communities in 100% of replications, with the exception of E, which was grouped within the Engagement cluster in 69% of iterations (Fig. 4).

### Multivariate multiple linear regression

**Figure 5:**
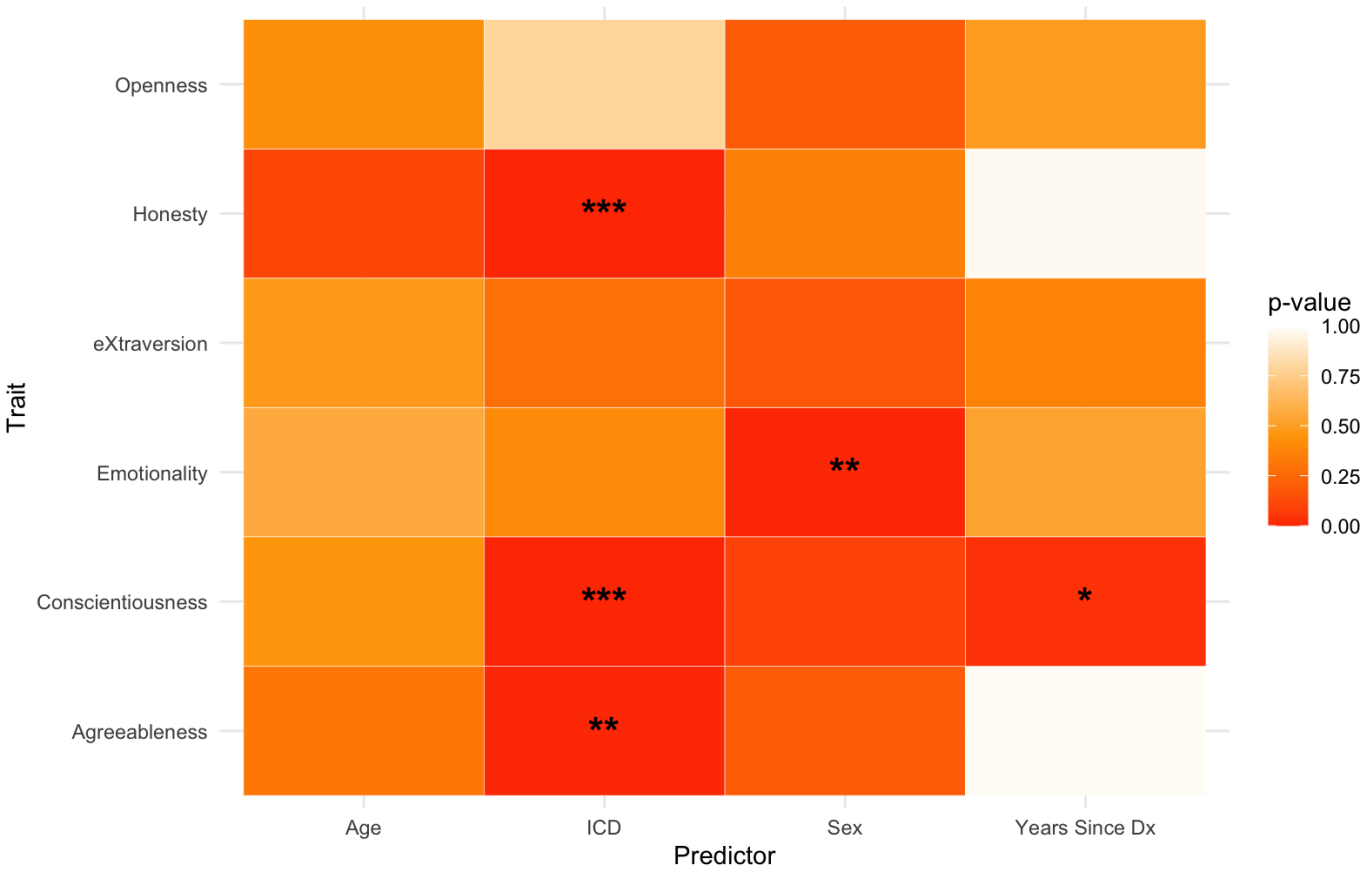
Heatmap showing *p*-values for the effects of demographic and clinical predictors on HEXACO traits. Asterisks indicate significant associations. * indicates *p* < .05, ** *p* < .01, *** *p* < .001.

The multivariate multiple regression model (sample size: 202 PD patients) examined the effects of Age, Sex, Years since Diagnosis, and Impulsive–Compulsive Disorder (ICD) symptom severity on the six HEXACO personality dimensions. Education (ISCED score) did not significantly improve model fit and was therefore excluded from the final model. Overall, the model indicated significant multivariate effects of Sex (Pillai = .132, F(180,6) = 4.84, *p* < .001) and ICD severity (Pillai = .137, F(180,6) = 5.13, *p* < .001) across traits. Figure 5 provides a visual summary of the significance patterns for each predictor–trait association.

#### Honesty-Humility

ICD symptom severity was a significant negative predictor of H (*β* = –.025, *p* < .001, R^2^adj = .112), indicating that higher levels of impulsive–compulsive behaviors were associated with lower sincerity, fairness, and modesty.

#### Emotionality

Sex had a significant effect on E (*β* = –.037, *p* = .003, R^2^adj = .033), with male participants scoring lower than female participants.

#### Extraversion

No significant effects were detected for any predictor.

#### Agreeableness

ICD score negatively predicted A (*β* = –.025, p = .001, R^2^adj = .045), suggesting that greater impulsive–compulsive symptoms were linked to reduced patience and tolerance.

#### Conscientiousness

C was influenced by both ICD severity and years since diagnosis. Specifically, higher ICD severity predicted lower C (*β* = –.037, *p* < .001), and so did longer disease duration (*β* = –.026, *p* = .03). Together, these effects explained a moderate proportion of variance (R^2^adj = .103), reflecting lower self-regulation and reliability among individuals with more pronounced impulsive–compulsive behaviors or longer disease course.

#### Openness

O was unaffected by any of the examined variables, indicating relative resilience of openness-related characteristics to disease progression or impulsive–compulsive symptoms.

## Discussion

This study aimed to investigate the structure of personality in individuals with PD and to explore how demographic and clinical variables—age, sex, education, years since diagnosis, and impulsive-compulsive tendencies—are associated with personality traits.

Personality structure was first examined using a network approach at the item level. The EGA approach revealed a seven-community solution that broadly aligned with the six HEXACO dimensions, while also highlighting subtle structural deviations. Specifically, clear and coherent communities emerged for the E, X, and C traits. H and A, on the other hand, did not form distinct clusters. Instead, items from both traits organized into two polarity-based communities—one comprising positively keyed A and H items (*e.g.*, *Peaceful, Loyal*) and the other the negative-valence ones (*e.g.*, *Litigious, Haughty*). This suggests a valence-driven reorganization in which H and A items clustered based on emotional tone rather than trait content. This pattern is consistent with prior findings highlighting structural and conceptual proximity between H and A, particularly in clinical or non-neurotypical populations (Ashton & Lee, 2007; Thielmann et al., 2022). Moreover, a recent study using the same model (HEXACO) and questionnaire (HAS) reported a similar valence-based reorganization in healthy older adults (Gobbo, Trapattoni & Romano, 2025), suggesting that this pattern reflects broader age-related personality dynamics rather than PD-specific alterations. A possible interpretation of this involves the well-documented positivity bias in aging (Carstensen, Isaacowitz & Charles, 1999; Mather & Carstensen, 2005). As people age, they tend to prioritize emotionally gratifying experiences and interpersonal harmony, placing greater value on prosocial and accommodating behaviors while de-emphasizing traits linked to criticism or norm enforcement. Consequently, descriptors such as *honest* and *kind* may converge in perceived meaning, just as arrogant and irritable may align through their negative emotional tone. This perceptual shift could lead conceptually distinct traits—such as Honesty–Humility and Agreeableness—to cluster together based on perceived valence, reflecting an age-related reorganization that favors diplomacy and conflict avoidance over bluntness or principled confrontation.

A second notable finding was the split of O items into two distinct communities. Namely, while most O items formed a coherent community, two adjectives—*Traditional* and *Conventional*—emerged as a separate cluster, corresponding to the Unconventionality facet. This bifurcation, though only modestly stable, may reflect the clinical salience of low unconventionality (high conventionality) in PD patients, a personality feature often identified as a hallmark of the Premorbid Parkinsonian Personality (Ishihara & Brayne, 2006b; Santangelo et al., 2018). According to this hypothesis, individuals with PD are often described as more conventional, orderly, and risk-averse even prior to diagnosis, raising the possibility that lower levels of unconventionality may be a core or early feature of PD. In this sense, the emergence of a separate cluster from these adjectives may reflect the salience of this trait facet within the PD population. Importantly, despite its potential relevance, the emergence of this separate community must be interpreted with caution. This is because the seven-community solution was replicated in 30% of bootstrap iterations—closely matched by the six-community solution at 26%—and item placement was inconsistent across resamples (the two Unconventionality items formed a separate community only 34% of the time), suggesting limited structural stability of this seventh community.

The overall sparsity of the network (density = 0.14; mean edge weight = 0.011) aligns with prior findings in personality network analysis, where items typically form tight intra-trait clusters while remaining conditionally independent across traits (Costantini et al., 2015; Costantini & Perugini, 2016; Gobbo, Trapattoni & Romano, 2025). This sparse yet structured organization supports the construct validity of the HEXACO model and underscores the utility of network models in clinical personality research.

A second analysis investigated personality structure at the trait level, exploring the organization of the six HEXACO dimensions into communities of traits more significantly related to one another than with the others. This approach revealed a robust two-community structure: one comprising H, A, and C (labeled as the Cooperativity–Integrity cluster) and the other formed by E, X, and O (Engagement cluster). This organization was highly stable across bootstrap iterations and maps well onto higher-order personality dimensions identified in Big Five research (Digman, 1997; DeYoung, Peterson & Higgins, 2002). The fact that this higher-order structure persists in a clinical population suggests that the fundamental architecture of personality may remain preserved in PD, even as certain dimensions—particularly those related to social functioning—undergo localized reorganization.

The second aim of this study was to examine how clinical (years since diagnosis, ICD score) and demographic variables (age, sex, education) relate to personality structure and individual HEXACO traits. The regression analysis indicated strong overall effects of ICD score and sex, a smaller effect of years since diagnosis, a modest contribution of age, and no effects of education. In line with previous findings (Raket et al., 2022), the distinct influences of age and disease duration likely reflect different mechanisms—general aging *versus* PD-specific progression—highlighting how both lifespan and neurodegenerative factors shape personality in PD.

At a broader level, trait-level analyses revealed distinct associations across dimensions. Higher ICD severity was linked to lower H, C, and A—the traits forming the Cooperativity–Integrity community—suggesting that greater impulsive–compulsive symptoms correspond to reduced self-regulatory and socially cooperative tendencies. These results align with previous findings connecting ICD symptoms to deficits in empathy, judgment, and executive control in PD (Poletti & Bonuccelli, 2012b; Voon et al., 2010). The correlation between C and the ICD score, in particular, may reflect underlying executive dysfunction, a common neuropsychological feature of PD, which likely contributes to difficulties with impulse control and goal-directed behavior. C was also negatively associated with years since diagnosis, as lower levels of C were observed in patients with longer disease duration, potentially indicating that maintaining planning, reliability, or goal-directed behavior becomes more difficult in the context of advancing motor, non-motor, and cognitive symptoms. Concerning Sex, this variable emerged as a significant predictor of E, with male participants showing lower scores in this personality trait. This pattern, consistent with findings in the general population (Lee & Ashton, 2020) may be amplified in PD patients by sex-related differences in emotional processing, coping styles, or help-seeking behavior—factors which could have implications for emotional well-being and treatment engagement. While causality cannot be established due to the cross-sectional nature of the data, this result may reflect both neurodegenerative and psychosocial aspects of PD’s long-term impact. By contrast, O and X were not significantly associated with any of the variables tested. This finding suggests that these traits may be relatively resistant to PD-related changes or, if differences from healthy populations exist, that they reflect premorbid personality dispositions—such as lower unconventionality within the O domain—rather than disease-related alterations.

Taken together, these findings highlight the clinical relevance of personality assessment in PD. Trait configurations—particularly those involving self-control, integrity, and emotionality—vary systematically with disease duration, impulsive-compulsive symptoms, and sex. Combined with the network results, these patterns point to both structural and functional shifts in personality that may inform early detection, patient profiling, and individualized care strategies in PD.

### Limitations

This study is not without limitations. First, its cross-sectional design prevents causal inferences about the relationships between personality traits and clinical variables, as well as conclusions about the premorbid nature of these traits. Second, personality data were collected through self-report questionnaires which, although valuable for capturing internal experiences and self-perceptions (Vazire, 2010), may be influenced by response biases or by PD-related cognitive deficits. Additionally, the online nature of data collection may have introduced selection effects, likely favoring participation by younger and more cognitively intact individuals. This sampling bias may have limited the representativeness of the sample and reduced the visibility of more severe behavioral or cognitive symptoms. The wide age range of participants also warrants consideration, as personality–disease associations might differ between younger and older individuals. While stratifying the sample by age (*e.g.*, under and over 65 years) was not feasible due to numerosity constraints, such an approach in future research could help clarify whether the observed effects reflect aging, disease progression, or their interaction.

Future studies should employ longitudinal designs to track personality changes over time, integrate informant or clinician reports to complement self-assessments, and include neurobiological, cognitive, motor, and medication-related measures to better elucidate the mechanisms linking personality and disease progression.

## Conclusions

This study investigated the structure and clinical correlates of personality traits in Parkinson’s disease using network-based and multivariate regression analysis approaches. The findings indicate that the overall HEXACO personality architecture is broadly maintained, with localized deviations reflecting subtle structural reorganization. The observed overlap between H and A likely reflects age-related positivity biases, while the bifurcation of Openness items underscores the salience of (un)conventionality within the PD personality profile. However, traits linked to cooperativity and integrity (H, A, and C) appear sensitive to PD-related factors such as disease duration and impulsive–compulsive symptoms.

At a broader level, these results suggest that personality in PD is shaped by both neurobehavioral and psychosocial processes, revealing selective changes in domains central to social functioning and behavioral control. Recognizing these configurations in clinical settings may help identify patients at higher risk for impulsive–compulsive behaviors, emotional dysregulation, or reduced treatment adherence. Incorporating personality assessment into multidisciplinary care could therefore promote more individualized management and improved quality of life.

##  Supplemental Information

10.7717/peerj.20725/supp-1 Supplemental Information 1 Raw dataset containing participants’ demographic information and the scores at the HAS and the QUIP-RS questionnaires.

10.7717/peerj.20725/supp-2 Supplemental Information 2 STROBE Checklist

10.7717/peerj.20725/supp-3 Supplemental Information 3 Questionnaire (English)

## Additional Information and Declarations

### Data Availability

The following information was supplied regarding data availability: The data and codes employed in this study are available at OSF: Vicentin, Stefano. 2025. “Structure of Personality in Parkinson’s Disease - SPARKS.” OSF. July 11. http://osf.io/rgtck.
