# Supplementary material for: The structure of personality in Parkinson’s disease and the effects of age, years since diagnosis, and impulsivity
Source: PeerJ. 2026 Feb 19;14:e20725. doi: 10.7717/peerj.20725 (PMC12925408; doi:10.7717/peerj.20725)
Supplement: Supplemental Information 3 [file peerj-14-20725-s003.docx]

**Questionnaire for Patients with Parkinson’s Disease**

Thank you for agreeing to participate in our research, titled *“The Structure of Personality in Parkinson’s Disease.”*
If you have been diagnosed with Parkinson’s Disease, we ask that you complete the questionnaire referring to yourself.
If instead you are a caregiver or acquaintance of someone with this diagnosis, please complete the questionnaire referring to that person.

The questionnaire is anonymous, and the data you provide will be used **exclusively in aggregate form and for research purposes**.
After reading and accepting the informational documents below, you will be presented with a series of questions and asked to complete questionnaires by following the instructions provided throughout.

Please remember that **there are no right or wrong answers** — we are only interested in your **personal opinion**.

**Study Participation Invitation**

You are invited to participate in the study *“The Structure of Personality in Parkinson’s Disease”* coordinated by the University of Padua.
Below, we provide information about the study and the potential use of your data so that you can make a **fully informed and voluntary decision** about participating.

**What is the purpose of this study?**

The aim of this study is to examine the **personality traits and cognitive autonomy** of individuals diagnosed with Parkinson’s Disease through the administration of a recently developed questionnaire: the **HEXACO Adjective Scale (HAS)**.

The questionnaire will be administered both to individuals with a Parkinson’s diagnosis and to their **family members or caregivers** (referred to as *Informant Reports*), who will complete the form referring to their relative with the diagnosis.

These data will then be compared with those of individuals with other neurological conditions (e.g., Alzheimer’s Disease) or without any such diagnosis, in order to highlight **differences and similarities related to different types of aging**.

In this way, we aim to improve understanding of personality traits and their changes associated with the onset of Parkinson’s Disease.

**Are you required to participate in the study?**

No, your participation is entirely voluntary.
Furthermore, you are **free to withdraw at any time** should you change your mind.

**Other Important Information**

This study will be conducted in accordance with the **ethical principles** outlined in the **Declaration of Helsinki** and the **Convention on Human Rights and Biomedicine (Oviedo Convention).**

**Who to contact for more information or clarification**

If you would like more information about this study, you can contact the researchers listed below:

- **Dr. Lavinia Chiriatti** – lavinia.chiriatti@studenti.unipd.it
- **Dr. Stefano Vicentin** – stefano.vicentin@unipd.it

### ****CONSENT TO DATA PROCESSING****

I, the undersigned, having read and understood the information provided above,
**give my consent to participate** in the research study titled “The Structure of Personality in Parkinson’s Disease”,
and **authorize the processing of personal data** necessary for carrying out the study/research project.

- I authorize
- I do not authorize

Can you tell us the exact diagnosis you were given?

________________________________________________________________

|  |
| --- |

Can you tell us approximately how many years ago the diagnosis was made?

________________________________________________________________

**IMPORTANT:** We ask you to create an anonymous and unique code through which we will store your data.
To do this, please write below:

- the **initials** of the first and last name of your relative/loved one with Parkinson’s Disease,
- their **date of birth**,
- and the letter **M** if male or **F** if female.

**Example:**
If your loved one’s name is Marta Rossi and she was born on July 2, 1965, write: **MR020765F**
If you are referring to Ugo Verdi, born on December 17, 1948, write: **UV171248M**

_____________________________________________________________

|  |
| --- |
|  |

How old are you?

_______________________________________________________________

**What is your highest level of education?**
(Primary school, middle school, high school, etc.)

________________________________________________________________

|  |
| --- |

**Are they currently admitted to any facility?**
If yes, which one?

**Below, we ask you to indicate how well each of the following adjectives (or short phrases) describes you, using the response scale provided.**
We understand that in many cases your answer may depend on the specific situation.
However, we ask that you respond based on how well each adjective describes you “on average” over the recent period, across different situations.

Please try to answer as sincerely as possible.
We remind you that your responses are **anonymous**, and no one — including your relatives or loved ones — will be able to trace your answers.

|  | It does not describe me at all | 2. | 3. | 4. | 5. | 6. | It completely describes me |
| --- | --- | --- | --- | --- | --- | --- | --- |
| Arrogant |  |  |  |  |  |  |  |
| Brave |  |  |  |  |  |  |  |
| Cheerful |  |  |  |  |  |  |  |
| Aggressive |  |  |  |  |  |  |  |
| Attentive |  |  |  |  |  |  |  |
| Appreciative of Art |  |  |  |  |  |  |  |
| Greedy |  |  |  |  |  |  |  |
| Emotional |  |  |  |  |  |  |  |
| Antisocial |  |  |  |  |  |  |  |
| Calm |  |  |  |  |  |  |  |
| Conscientious |  |  |  |  |  |  |  |
| Unnterested in Art |  |  |  |  |  |  |  |
| Dishonest |  |  |  |  |  |  |  |
| Fragile |  |  |  |  |  |  |  |
| Outgoing |  |  |  |  |  |  |  |
|  | It does not describe me at all | 2. | 3. | 4. | 5. | 6. | It completely describes me |
| Hot-tempered |  |  |  |  |  |  |  |
| Diligent |  |  |  |  |  |  |  |
| Conventional |  |  |  |  |  |  |  |
| Loyal |  |  |  |  |  |  |  |
| Emotionally detached |  |  |  |  |  |  |  |
| Extraverted |  |  |  |  |  |  |  |
| Quarrelsome |  |  |  |  |  |  |  |
| Disorganized |  |  |  |  |  |  |  |
| Curious |  |  |  |  |  |  |  |
| Hypocritical |  |  |  |  |  |  |  |
| Unflappable |  |  |  |  |  |  |  |
| Introverted |  |  |  |  |  |  |  |
| Peaceful |  |  |  |  |  |  |  |
| Absent--minded |  |  |  |  |  |  |  |
| Innovative |  |  |  |  |  |  |  |
|  |  |  |  |  |  |  |  |
|  | It does not describe me at all | 2. | 3. | 4. | 5. | 6. | It completely describes me |
| Rude |  |  |  |  |  |  |  |
| Faithful |  |  |  |  |  |  |  |
| Oversensitive |  |  |  |  |  |  |  |
| Quiet |  |  |  |  |  |  |  |
| Patient |  |  |  |  |  |  |  |
| Inaccurate |  |  |  |  |  |  |  |
| Intellectual |  |  |  |  |  |  |  |
| Honest |  |  |  |  |  |  |  |
| Fearful |  |  |  |  |  |  |  |
| Sociable |  |  |  |  |  |  |  |
| Overbearing |  |  |  |  |  |  |  |
| Reckless |  |  |  |  |  |  |  |
| Uncreative |  |  |  |  |  |  |  |
| Truthful |  |  |  |  |  |  |  |
| Confident |  |  |  |  |  |  |  |
|  | It does not describe me at all | 2. | 3. | 4. | 5. | 6. | It completely describes me |
| Solitary |  |  |  |  |  |  |  |
| Beligerent |  |  |  |  |  |  |  |
| Inconsistent |  |  |  |  |  |  |  |
| Original |  |  |  |  |  |  |  |
| Snobbish |  |  |  |  |  |  |  |
| Emotionally stable |  |  |  |  |  |  |  |
| Shy |  |  |  |  |  |  |  |
| Tolerant |  |  |  |  |  |  |  |
| Organized |  |  |  |  |  |  |  |
| Unoriginal |  |  |  |  |  |  |  |
| Humble |  |  |  |  |  |  |  |
| Vulnerable |  |  |  |  |  |  |  |
| Lively |  |  |  |  |  |  |  |
| Tranquil |  |  |  |  |  |  |  |
| Precise |  |  |  |  |  |  |  |
| Traditionalist |  |  |  |  |  |  |  |

You will now be presented with the same adjectives you just saw.

This time, however, we ask you to indicate how well each of the following adjectives describes you compared to ten years ago.

|  | It does not describe me at all | 2. | 3. | 4. | 5. | 6. | It completely describes me |
| --- | --- | --- | --- | --- | --- | --- | --- |
| Arrogant |  |  |  |  |  |  |  |
| Brave |  |  |  |  |  |  |  |
| Cheerful |  |  |  |  |  |  |  |
| Aggressive |  |  |  |  |  |  |  |
| Attentive |  |  |  |  |  |  |  |
| Appreciative of Art |  |  |  |  |  |  |  |
| Greedy |  |  |  |  |  |  |  |
| Emotional |  |  |  |  |  |  |  |
| Antisocial |  |  |  |  |  |  |  |
| Calm |  |  |  |  |  |  |  |
| Conscientious |  |  |  |  |  |  |  |
| Unnterested in Art |  |  |  |  |  |  |  |
| Dishonest |  |  |  |  |  |  |  |
| Fragile |  |  |  |  |  |  |  |
| Outgoing |  |  |  |  |  |  |  |
|  | It does not describe me at all | 2. | 3. | 4. | 5. | 6. | It completely describes me |
| Hot-tempered |  |  |  |  |  |  |  |
| Diligent |  |  |  |  |  |  |  |
| Conventional |  |  |  |  |  |  |  |
| Loyal |  |  |  |  |  |  |  |
| Emotionally detached |  |  |  |  |  |  |  |
| Extraverted |  |  |  |  |  |  |  |
| Quarrelsome |  |  |  |  |  |  |  |
| Disorganized |  |  |  |  |  |  |  |
| Curious |  |  |  |  |  |  |  |
| Hypocritical |  |  |  |  |  |  |  |
| Unflappable |  |  |  |  |  |  |  |
| Introverted |  |  |  |  |  |  |  |
| Peaceful |  |  |  |  |  |  |  |
| Absent--minded |  |  |  |  |  |  |  |
| Innovative |  |  |  |  |  |  |  |
|  |  |  |  |  |  |  |  |
|  | It does not describe me at all | 2. | 3. | 4. | 5. | 6. | It completely describes me |
| Rude |  |  |  |  |  |  |  |
| Faithful |  |  |  |  |  |  |  |
| Oversensitive |  |  |  |  |  |  |  |
| Quiet |  |  |  |  |  |  |  |
| Patient |  |  |  |  |  |  |  |
| Inaccurate |  |  |  |  |  |  |  |
| Intellectual |  |  |  |  |  |  |  |
| Honest |  |  |  |  |  |  |  |
| Fearful |  |  |  |  |  |  |  |
| Sociable |  |  |  |  |  |  |  |
| Overbearing |  |  |  |  |  |  |  |
| Reckless |  |  |  |  |  |  |  |
| Uncreative |  |  |  |  |  |  |  |
| Truthful |  |  |  |  |  |  |  |
| Confident |  |  |  |  |  |  |  |
|  | It does not describe me at all | 2. | 3. | 4. | 5. | 6. | It completely describes me |
| Solitary |  |  |  |  |  |  |  |
| Beligerent |  |  |  |  |  |  |  |
| Inconsistent |  |  |  |  |  |  |  |
| Original |  |  |  |  |  |  |  |
| Snobbish |  |  |  |  |  |  |  |
| Emotionally stable |  |  |  |  |  |  |  |
| Shy |  |  |  |  |  |  |  |
| Tolerant |  |  |  |  |  |  |  |
| Organized |  |  |  |  |  |  |  |
| Unoriginal |  |  |  |  |  |  |  |
| Humble |  |  |  |  |  |  |  |
| Vulnerable |  |  |  |  |  |  |  |
| Lively |  |  |  |  |  |  |  |
| Tranquil |  |  |  |  |  |  |  |
| Precise |  |  |  |  |  |  |  |
| Traditionalist |  |  |  |  |  |  |  |

**In the next questionnaire, we ask you to answer questions about your degree of impulsivity.**
Please try to answer as sincerely as possible. We remind you that your responses are **anonymous**, and no one will be able to trace your answers.

**How often do you have thoughts related to the following behaviors** (for example, how difficult it is to keep these thoughts out of your mind, or how often you feel guilty about them)?

|  | Never | Rarely | Sometimes | Often | Very often |
| --- | --- | --- | --- | --- | --- |
| Gambling |  |  |  |  |  |
| Sex |  |  |  |  |  |
| Shopping |  |  |  |  |  |
| Eating |  |  |  |  |  |
| Engaging in activities or hobbies |  |  |  |  |  |
| Repetition of simple activities |  |  |  |  |  |
| Taking specific medications for Parkinson’s disease |  |  |  |  |  |

**Do you feel the need or desire to engage in the following behaviors that you perceive as excessive or stressful** (including feeling restless or irritable when you are unable to carry them out)?

|  | Never | Rarely | Sometimes | Often | Very often |
| --- | --- | --- | --- | --- | --- |
| Gambling |  |  |  |  |  |
| Sex |  |  |  |  |  |
| Shopping |  |  |  |  |  |
| Eating |  |  |  |  |  |
| Engaging in activities or hobbies |  |  |  |  |  |
| Repetition of simple activities |  |  |  |  |  |
| Taking specific medications for Parkinson’s disease |  |  |  |  |  |

**Do you have difficulty controlling the following behaviors**
(for example, a tendency to increase their frequency over time or difficulty in reducing or stopping any of the following behaviors)?

|  | Never | Rarely | Sometimes | Often | Very often |
| --- | --- | --- | --- | --- | --- |
| Gambling |  |  |  |  |  |
| Sex |  |  |  |  |  |
| Shopping |  |  |  |  |  |
| Eating |  |  |  |  |  |
| Engaging in activities or hobbies |  |  |  |  |  |
| Repetition of simple activities |  |  |  |  |  |
| Taking specific medications for Parkinson’s disease |  |  |  |  |  |

**Do you engage in activities with the purpose of maintaining the following behaviors**
(for example, hiding what you are doing, lying, hoarding items, asking for loans, accumulating debt, stealing, or being involved in illegal activities)?

|  | Never | Rarely | Sometimes | Often | Very often |
| --- | --- | --- | --- | --- | --- |
| Gambling |  |  |  |  |  |
| Sex |  |  |  |  |  |
| Shopping |  |  |  |  |  |
| Eating |  |  |  |  |  |
| Engaging in activities or hobbies |  |  |  |  |  |
| Repetition of simple activities |  |  |  |  |  |
| Taking specific medications for Parkinson’s disease |  |  |  |  |  |
